# Mental Health Care for Patients with Opioid Use Disorder and Methamphetamine Use in Methadone Maintenance Treatment: Experiences and Perspectives

**DOI:** 10.1007/s11469-025-01616-w

**Published:** 2025-12-19

**Authors:** Huyen Pham, Chunqing Lin, Diep Bich Nguyen, Larissa J. Mooney, Giang Minh Le, Steven J. Shoptaw, Hai Van Truong, Yih-Ing Hser

**Affiliations:** 1 Department of Psychiatry and Biobehavioral Sciences, Semel Institute for Neuroscience and Human Behavior, University of California, 10911 Weyburn Avenue, Suite 200, Los Angeles, CA 90024, USA; 2 School of Preventive Medicine and Public Health, Hanoi Medical University, Hanoi, Vietnam; 3 VA Greater Los Angeles Healthcare System, Los Angeles, CA 90073, USA; 4 Department of Family Medicine, University of California, Los Angeles, CA 90024, USA

**Keywords:** Psychiatric comorbidity, Methadone maintenance treatment, Substance use disorders, Integrated care, Vietnam

## Abstract

Untreated co-occurring mental health disorders (MHD) and substance use disorders (SUD) are associated with negative treatment outcomes. This study explored the experiences and perspectives of patients with opioid use disorder (OUD) who were using methamphetamine while undergoing methadone maintenance treatment (MMT) regarding barriers and facilitators to accessing MHD services. We conducted 30 in-depth interviews with MMT participants with varying levels of MHD severity, assessed using the 21-item Depression, Anxiety, Stress Scale (DASS-21). Barriers to seeking MHD services included lack of awareness, fear of additional stigma and extra burden to families, unaddressed poly-substance use, and lack of specialized professionals. Benefits of integrating MHD services into MMT clinics included improved diagnosis, streamlined referrals, and reduced logistical challenges. Key facilitators identified included supportive family members, healthcare professionals, and digital platforms. Integrating MHD care into MMT, emphasizing family involvement, staff training, and digital platforms, holds promise to improve MHD diagnosis, referral, and treatment.

Untreated co-occurring mental health disorders (MHDs) in individuals with substance use disorders (SUDs) are linked with poor outcomes including treatment discontinuation and reduced quality of life (Cacciola et al., 2001; Carpentier et al., 2009; Compton III et al., 2003; Strain, 2002). Managing both conditions is complex as symptoms can mask or exacerbate each other, complicating diagnosis and care (Iqbal et al., 2019). Barriers to MHD care include a global shortage of trained health professionals, particularly in low- and middle-income countries (WHO, 2015), fragmented healthcare systems, limited clinical guidelines, resource constraints, and high staff burnout and turnover (California Health Care Foundation, 2021; Javed et al., 2021; M. X. Nguyen et al., 2019a, 2019b; Oviedo et al., 2023). Social factors such as poverty, unemployment, and stigma further hinder access to care (Agterberg et al., 2020; Aguirre Velasco et al., 2020; Choudhry et al., 2016; El Hayek et al., 2024; Javed et al., 2021; Kuek et al., 2023). In many countries, cultural beliefs also shape treatment-seeking behaviors; for instance, mental illness is often seen as “immoral” and substance use as a societal threat, influencing care and policy (Li et al., 2020; K. Trang et al., 2021).

Vietnam, heavily affected by the opioid and HIV epidemics, has expanded methadone maintenance treatment (MMT) in response (Ngoc et al., 2013). However, MMT patients in Vietnam are vulnerable to MHD comorbidity (Mughal et al., 2021; L. H. Nguyen et al., 2017), which is associated with poorer treatment outcomes (Ali et al., 2017; Cacciola et al., 2001; Carpentier et al., 2009; L. H. Nguyen et al., 2017). As in other low- and middle-income countries, Vietnam faces a shortage of MHD resources, with MHD medications limited to few facilities (M. X. Nguyen et al., 2019a, b; Vuong et al., 2011). MHD services remain separate from MMT settings, creating gaps in comprehensive care (H. V Tran et al., 2022a, b).

Integrated care models, including single- and multi-facility integration, integration through task-shifting, and system integration (Valentijn et al., 2013), have shown promise in addressing the barriers to care (Chuah et al., 2017; Flexhaug, 2012; Glover-Wright et al., 2023; Richardson et al., 2020). Although various integrated care models for comorbid SUD and MHD have been implemented and evaluated in Western and other high-income settings (Richardson et al., 2020), little is known about how an integrated care model functions in Eastern countries including Vietnam, where cultural norms and perspectives may differ substantially. While it is important to examine participant perspectives to provide person-centered care, research on their insights regarding integrated care models remains limited (Richardson et al., 2020; Silva & Andersson, 2021).

This paper presents a formative study among MMT patients with co-occurring methamphetamine use and MHD in Vietnam. We sought to understand their experiences and perspectives of comorbid MHD and SUD, MHD symptoms and care, perceptions of integrated care and factors influencing MHD treatment seeking. Findings will inform culturally sensitive health service modalities to improve access and quality of care in resource-limited settings.

## Method

### Study Participants

Our study was built upon a clinical trial, which tested the effectiveness of various combinations of evidence-based interventions for methamphetamine use among patients undergoing methadone treatment for OUD (Giang et al., 2022). The parent study used the 21-item Depression, Anxiety, and Stress Scale (DASS-21) to measure the severity of symptoms (Giang et al., 2022; T. D. Tran et al., 2012). We used the DASS-21 scores to categorize patients’ severity levels, as follows: Depression—normal (0–9), mild (10–13), moderate (14–20)/severe (≥ 21); Anxiety—normal (0–7), mild (8–9), moderate (10–14)/severe (≥ 15); stress—normal (0–14), mild (15–18), moderate (19–25)/severe (≥ 26). Recruitment prioritized diversity and adequate representation across different MHD severity levels, sex, and age.

Between October and December 2023, we conducted 30 in-depth interviews among participants from the parent study, who were aged 18 or older with varying levels of MHD. We continued recruiting and interviewing participants until we reached thematic saturation, defined as the point at which no substantially new themes emerged from successive interviews. To recruit participants, the parent study’s coordinators invited participants who consented to share their information with a follow-up study via their written consent forms. The coordinators distributed information flyers, recruitment letters, or word-of-mouth invitations to potential participants. Our recruiting staff discussed study procedures, reviewed participants’ rights, risks and benefits, and emphasized that participation was entirely voluntary. Oral informed consent was obtained before each interview. The study was approved by the Institutional Review Boards at University of California, Los Angeles (IRB#23–000542) and Hanoi Medical University (IRB-VN01.001/IRB0003121).

### Data Collection

The interviews were conducted in Vietnamese by native researchers who were trained and experienced in qualitative research. Interviews took place privately in MMT clinic offices. The semi-structured interview guidelines covered the following topics: (1) general information about health and family, (2) drug use and methadone treatment, (3) perceptions of mental health and experience with any MHDs, (4) perceptions and experience of MHD treatment including regular MHD screening and linkage to care provided by their methadone providers or other service providers, (5) facilitators and barriers of obtaining MHD diagnoses and treatment in patients’ living areas, and (6) perceived integration of MHD services into MMT. Regarding perceptions of mental health, we inquired whether the participants were familiar with MHD (“Roi loan suck hoe tam than”), depression (“Tram cam”), and anxiety disorders (“Roi loan lo au”). If they indicated unfamiliarity, interviewers offered explanations of these conditions to understand the participants’ perspectives and alternative terms used to describe them. For instance, MHD was described as “mental health disorders affecting mood, cognition, and behavior, often leading to disruptions in eating or sleeping patterns and withdrawal from social interactions”; depression as “prolonged feelings of sadness accompanied by loss of interest in daily activities and previously enjoyed hobbies”; and anxiety disorders as “excessive and debilitating worry interfering with daily life, often accompanied by physical symptoms such as rapid heartbeat, dizziness, and sweating.” No personal identifying information was asked for the interviews. Each interview lasted 60–90 min. All participants consented to have their interview audio recorded. The participants received 360,000 VND (approximately 15 USD) as compensation for their time and travel expenses.

### Data Analysis

The audiotapes of the in-depth interviews were transcribed verbatim. We applied a reflexive thematic analysis (Braun & Clarke, 2022) using a hybrid approach: initial codes were informed by our pre-developed interview guide, and additional themes were iteratively derived from participants’ responses. The researchers summarized the main findings after each interview and noted additional questions for next interviews. Data collection and analysis occurred iteratively, allowing the team to monitor emerging concepts and assess saturation throughout the process. We developed a set of codes based on the interview guide before examining the data. The codes were applied to the transcripts and notes from the interviewers. The codebook was modified based on themes that emerged from the transcripts and notes throughout the coding procedure. The data segments were organized into coding categories using ATLAS.ti. Researchers from both countries participated in the coding process. To ensure inter-coder reliability, the researchers jointly coded 10% of the transcripts, independently read the transcripts, discussed analysis and coding, and discussed divergence until reaching agreement. We refined the definition of codes several times during the process of analysis, and the application of codes was updated accordingly. Based on repeated reviews of coded data and team-based discussion, we confirmed that thematic saturation had been reached when no new themes or subthemes emerged. Themes relevant to comorbid MHD and SUD perceptions, MHD care experience, barriers and facilitators to addressing MHD in MMT clinics were developed, with corresponding quotations presented in the results section.

## Results

The majority of participants were male (83%), while 17% were female. The average age was 42 years, with 53% of participants falling in the 40–49 age range, 37% in the 30–39 range, and 10% aged 50 or older. According to the DASS-21 assessment, 63% of participants reported experiencing anxiety, 40% reported experiencing depression, and 20% reported experiencing stress. Most participants were employed, with 77% working full time and 13% working part-time. Over 60% had not completed high school, and approximately 40% were single or divorced. (see Table 1).

## Patient Perspectives of Comorbid MHD and SUD

Participants frequently attributed their MHD to their past prolonged use of opioids or heroin, exacerbated by excessive methamphetamine use. A 42-year-old male patient with OUD undergoing MMT treatment reflected on this phenomenon. He noted that both he and other MMT patients believed their prolonged poly-substance use had significantly impaired brain function.

Because most of our generation, those who use drugs, mostly end up damaging their brains, their nervous system. Crystal meth, marijuana, various types, they ruin your mind completely.

(42-year-old, male MMT patient with severe anxiety, severe depression, and mild stress)

Participants also stressed the influence of the interplay of stigma (see Table 2) and self-stigma. They described how societal judgment, coupled with internalized shame, exacerbated their struggles, often accelerating mental health deterioration. While some participants noted individual differences in coping with these challenges, the impact of stigma appeared to be broadly harmful. As a 48-year-old, male MMT patient explained:

[…]this guy is an addict, he can never quit, he’s addicted to heroin, his nerves are ruined, stay away from him, don’t get close to him and catch the disease. Those are the sarcastic remarks of society, of the people around, pushing oneself to drift along that stream faster. Those words make one deteriorate faster. Because when someone is mentally ill, they are very sensitive to words. And you who have studied, you know. Sometimes a punch, a kick, a slap doesn’t do much. But just one sentence, ready to make someone collapse. Ready to make someone’s nerves spiral back immediately. That’s very scary.

(48-year-old, male MMT patient with mild anxiety)

When asked how they managed such situations, participants disclosed that they used substances as a way to cope with MHD symptoms. For instance, one participant described turning to drug use to deal with feelings of depression and related symptoms, unaware that this behavior could contribute to addiction later on.

…it’s very depressing… and I thought like this, later I thoughtthat’s depression, just this kind of situation… “Just using [substance] for now, think about it later.” Hoping that drug would solve everything, at that time Ididn’t think about getting addicted again, just used to forget all those annoyances.

(44-year-old, male MMT patient with severe anxiety)

## Experience of Comorbid MHD Symptoms and MHD Treatments

Overall, participants in MMT demonstrated better understanding of depression as a disorder than of “anxiety” as a disorder. Common phrases used to describe depressive symptoms include alone (“Mot minh”), reserved (“Khong muon noi chuyen voi ai het”), disenchanted (“Chan chuong”), dreary (“Buon chan”), suicide “Tu tu”, inexorable (“Khong the vuot qua duoc”), and apathetic (“Khong thich gi nua”). On the other hand, common phrases describing anxiety-related symptoms include anxious (“Lo au”), immersed (“Xoay vao day”), unending (“Khong dut duoc”), unfounded (“Buon lo vo co”), and insomnia (“Mat ngu”).

Sixty percent (18 participants) shared their own depressive symptoms, and 30% (10 participants) shared their own anxiety related symptoms (Table 3). Thirteen percent of individuals with severe levels of stress also exhibited severe levels of depression and anxiety. They mostly highlighted their depression or anxiety symptoms, but also emphasized their persistent insomnia (“Mat ngu trien mien”) and distress (“Buon buc”) symptoms. One participant experiencing severe levels of depression, anxiety, and stress had a suicide attempt in the past.

Only three out of 18 participants (18%) who experienced severe depression symptoms ever sought treatment. None of the participants who experienced symptoms of anxiety ever searched for related treatment. None of the participants who experienced stress-related symptoms ever sought treatment to manage their stress.

Individuals sought MHD treatment primarily due to severe symptoms that involved harm to others or themselves (i.e., suicidal thoughts or actions). In such cases, family members or local authorities were the ones to send these serious cases to MHD treatment settings. However, many family members associated MHD exclusively with substance dependence and preferred that their loved ones seek treatment at a rehabilitation center for SUD instead. One participant shared that despite experiencing MHD for nearly a month, he actually sought a treatment to fulfill his family wishes, as they believed his MHD was solely a result of his SUD.

Back then, it was my parents and my sister who encouraged me and also took me to undergo detox… in the rehab center. That time was when I went through detox in the rehab center…

(40-year-old, male MMT participant, severe anxiety, severe depression, severe stress)

### Barriers to Access MHD Services

Participants also highlighted challenges including lack of MHD awareness, fear of additional stigma and discrimination, concerns about burdening their families by acknowledging MHD symptoms, discontinuation of MHD treatment due to other unaddressed SUD comorbidities like methamphetamine use disorder, and the lack of specialized MHD care.

#### Lack of Awareness of MHD Symptoms

“I did not know the symptoms I had were MHD at that time” (43-year-old, female, MMT participant with severe anxiety, mild depression, stress). Many participants shared that they did not seek MHD care because of their lack of awareness of MHD symptoms. Some perceived their MHD as normal or mild and so did not seek MHD treatment. Another reason was the fear that MHD treatment and medication might worsen their symptoms. Specifically, 30% reported witnessing or experiencing negative MHD treatment outcomes, whereas only 13% witnessed positive outcomes.

Oh, like the story I told, and also a friend of mine who, after using medication, was told that sometimes going to those mental institutions, sometimes [they were] not really crazy at first but becoming crazier because of taking too much medication.

(38-year-old, female MMT participant with severe anxiety and mild depression)

#### Fear of Stigma and Discrimination Against MHD and SUD Comorbidities

Another challenge to seeking any MHD services is the fear of dual stigma and discrimination to co-occurring MHD and SUD. Some participants were particularly concerned that society would associate their MHD with their SUD, especially methamphetamine use. For example, one participant shared that the prevalent belief that mental illness associated with synthetic stimulant use led to stigma and self-stigmatization. People often assumed that symptoms were the result of drug use rather than a medical condition causing prejudice, insecurity and rejection.

Because people think these individuals are not actually suffering from a medical condition caused by birth or accidents. They believe these individuals are using drugs. It’s because of using crystal meth or synthetic drugs that they exhibit such symptoms, which leads to prejudice, insecurity, and rejection.

(45-year-old, male MMT participant with severe anxiety, depression, and stress)

#### Those Suspecting MHD Hide Symptoms to Spare Family from Sadness

Many participants shared that their family members had already suffered from their prolonged substance use and SUD. Therefore, seeking MHD diagnosis and treatment would further burden their families and loved ones. To spare their families additional distress, they chose to hide their symptoms or not seek MHD treatment.

In reality, I don’t discuss much about the issue with my family because I have to handle it myself. Now, my mother, my wife, have suffered for so many years, and if I keep talking about my own problems, it only adds to their worries. So I think it’s best not to say anything; I can handle it because, in reality, it’s just temporary. So, I just come home and talk about feeling sick, feeling this way, feeling that way, and it makes them worried. When my family worries a little, I worry more. When I feel down, I pick myself up again. Sometimes, I just have to hide and can’t speak up.”

(44-year-old, male MMT participant with severe anxiety)

#### Other SUD Comorbidities Including Methamphetamine Use Disorder

Participants also indicated that challenges to continuing MHD treatment include other unaddressed SUDs, such as methamphetamine use disorder. As one 47-year-old male MMT participant with severe anxiety, mild depression, and normal stress stated, those who continue using substances often no longer prioritize treatment.

They don’t want treatment… but for anyone who doesn’t want to use anymore, the mindset is just about seeking treatment. For those who continue using[methamphetamine], treatment is no longer important.

(47-year-old, male MMT participant with severe anxiety and mild depression)

#### Lack of Specialty MHD Treatment

One reason participants avoided seeking MHD care was their perception that MMT healthcare staff lacked expertise in MHD treatment. As one participant explained his decision not to seek care despite experiencing symptoms:

That’s right, they don’t know much about it. They only specialize in methadone treatment, so they can’t know everything. There’s no point in asking too much. (38-year-old, male MMT participant with severe anxiety, severe depression, severe stress).

Some participants emphasized the challenges of managing the MHD care given their already unstable mental state, highlighting the need for highly experienced professionals. They believed that addressing MHD among MMT patients would require specialized doctors or additional training for MMT healthcare staff. Additionally, participants stressed that these providers should possess qualities they described as enthusiastic, sincere, and experienced.

You need people like skilled doctors with expertise to do it… In mental health treatment and methadone treatment. How can nurses manage it? I just think simply that to be able to do it, first you need a team of staff who are truly enthusiastic and sincere when dealing with these addicts. Because dealing with these addicts is very difficult. Our mental state is already very unstable, not deliberately, but really not stable. So, at very least, you need an extremely experienced team to do this work

(38-year-old, male MMT participant with severe anxiety, depression, and stress)

### Addressing MHD Comorbidity in Patients in MMT Treatment: Facilitators and Opportunities

Facilitators for addressing MHD comorbidity in the MMT clinic context included the widespread use of online platforms to assess MHD and search for MHD care, family support, and factors related to the integration of MHD services into MMT clinics. Participants identified key integration factors, such as MMT healthcare staff assessing MH symptoms, connecting patients to the appropriate care, and providing convenience in terms of transportation, finances, and time.

#### Online Platform for Identifying MHD Symptoms and Services

Participants highlighted the significant role of technology and the internet in learning about meaning of symptoms and locating available MHD services. As a participant explained that when she experienced symptoms of mental health distress, she hesitated to share with anyone else, instead she used her phone to search online. Upon comparing her experiences to the listed depressive symptoms, she shared that most of them—about 8 out of 10—seemed to match what she was going through.

I did not dare tell anyone. I just looked into them [the symptoms]. I did some research online and, on my phone, and when I saw some of the symptoms, I felt like 8 out of 10 of them applied to me.

(43-year-old, female MMT participant with severe anxiety, mild depression, mild stress)

Many patients possessed a good understanding of the availability of MHD treatments and how to find them, thanks to advancements on the internet and technology. Some participants were able to provide specific examples of places offering MHD treatments.

In general, I think there aren’t any difficulties because nowadays, information about healthcare services are more accessible than before, it’s easier. Now, you go online, search for something, and immediately find the answer to that issue. Or if it’s something you’re curious about… on your phone, then they… point out those points, it’s not difficult. It’s easy, there’s nothing hard to find.

(43-year-old, male MMT participant with severe anxiety)

#### Family Support

Participants consistently highlighted the importance of support from family members, especially spouses or those living in the same household. The reasons for the importance of family support were enumerated as follows: (1) family members were often the first to notice changes related to MHD, such as alterations in speech patterns and behaviors, which patients themselves might not recognize or acknowledge as symptoms; (2) family support could significantly enhance the effectiveness of MHD treatment and accelerate recovery, whereas without it, the recovery process could be prolonged; and (3) financial support from families could help cover the costs of medications for MHD or assist participants in getting back to work (e.g., purchasing a motor vehicle for shipping services), thereby fostering a sense of well-being.

I think, obviously, family will definitely be more important. Yes, because in this mental health issue, usually, those close to us will notice it first compared to ourselves. When we have something unusual, we often don’t notice it ourselves. But those who live close to us, they see and notice it.

(35-year-old, male MMT participant with severe anxiety and mild depression)

#### Healthcare Staff at MMT Clinics Foster Mental Health Dialogue

Participants expressed that integrating MHD services with MMT clinics could offer significant benefits for several reasons. First, MMT clinics create an environment where patients can openly discuss their mental health concerns, receiving potential support from healthcare professionals, and may find support and understanding among other MMT patients. Initially, many patients expressed that healthcare providers were observant of any symptoms they might be experiencing, sometimes proactively scheduling consultations. Patients felt comfortable sharing various aspects of their well-being with healthcare providers, including physicians and counselors, ranging from minor concerns such as poor appetite or sleep disturbances to more complex issues affecting their thought processes. Patients emphasized the importance of feeling listened to regarding their mental health concerns, and appreciated the advice and support provided by healthcare staff, whether through facilitating MHD diagnoses or connecting them with relevant services.

Yes. Like a doctor [specific name] and also you guys at [specific name] University…I told my friends that my “joy” is being doubled. That emotional shock is one. Crystal meth shock is another. So, at the same time, you’re hit with both. That’s the noteworthy thing. So, you guys were worried. You guys kept contacting me for continuous counseling, for check-ups.

(48-year-old, male MMT participant with mild anxiety)

Integrating MHD services into MMT clinics offers the advantage of potentially linking patients to appropriate care. As a MMT participant below indicated, the integration allows patients to seek advice from doctors, receive attention for their concerns, and potentially get timely interventions if serious issues are detected.

For example, if by any chance I have any issues, I can ask the doctors here for advice, inquire about my concerns. Secondly, if by any chance I have any problems, usually they will pay attention to it. And if that issue of mine is serious, who knows, the doctor might notice and intervene to help me.

(35-year-old, male MMT participant with severe anxiety and mild depression)

#### Convenience Regarding Transportation, Finances, and Time

Finally, convenience regarding transportation, finances, and time is highlighted as a significant benefit of receiving integrated services at MMT clinics. As explained by a participant below, having all necessary care in one location eliminates the need for multiple trips and appointments. This setup not only saves time and money but also ensures comprehensive care including mental, emotional, and psychological counseling alongside medication management.

Those who come here [methadone clinic] will be seen to approach the best, most caring care for the condition they are suffering from, without having to go far. There’s no need to go to different places for examination. It’s all right here. One-stop shopping for everything. When I come to take methadone, I also get checked up. Wherever the illness is, the doctor dispenses medication there. I take methadone every day and also take my other medication. It’s very convenient. Convenient for both transportation and finances. I think it’s best to have a clinic like I mentioned, counseling on mental, emotional, and psychological matters for psychiatric patients at this center. That’s always good.

(48-year-old, male MMT participant with mild anxiety)

## Discussion

This study enhances the understanding of patient perspectives on MHD care for co-occurring MHD and SUD from the insights from MMT patients with co-occurring methamphetamine use and different levels of MHD severity. This study provides an in-depth understanding of their experiences with MHD symptoms and the barriers to accessing MHD care. Our findings also highlight facilitators and opportunities for integrated care models in MMT clinics to address MHD comorbidity.

Results from this study indicate that major barriers to seeking MHD care include lack of awareness of MHD symptoms along with distrust in MHD treatment and medications. While the finding of lack of awareness of MHDs in general is consistent with previous studies (Lin et al., 2023; M. X. Nguyen et al., 2019a, b; Novak et al., 2019), our participants demonstrated a better understanding of depression symptoms as being an MHD than symptoms of anxiety disorders. This is concerning given that anxiety disorders are common among individuals with SUD and linked to poorer SUD treatment outcomes (Carpentier et al., 2009; Lai et al., 2015; Zhu et al., 2021). Notably, more than 50% of our participants had moderate to severe DASS-21defined anxiety disorders, and although ten participants could describe their experiences of anxiety-related symptoms, none sought care. Indeed, participants typically sought MHD care only when experiencing severe symptoms that posed a risk of self-harm or harming others. They were often referred by family members or local authorities. Future efforts should focus on raising awareness of common MHD problems, including education to dispel myths about MHD treatment and medications among MMT patients and family members. Additionally, MHD interventions should improve MHD screening, diagnoses, and referral at MMT clinics.

Our study offers novel insights into the decision-making processes of individuals with co-occurring SUD and MHD regarding MHD care. While participants were aware of their likely MHD symptoms, many deliberately avoided seeking MHD care due to prior experiences of severe stigma as users of heroin or other substances, both before and during MMT treatment. Importantly, their decision to engage in MMT was largely driven by a desire to restore a sense of normalcy, overcome heroin addiction, and preserve stability within the family, particularly financial stability (T. T. Nguyen et al., 2019a, b). The added label of an MHD diagnosis was perceived as a threat to their efforts, leading many to avoid clinical diagnoses, referrals, and treatment despite experiencing symptoms. Alarmingly, some participants resorted to use of other substances to self-manage their mental health symptoms, a pattern that may exacerbate MHDs and create additional treatment challenges (Gold et al., 2020). While previous studies highlighted the role of stigma and discrimination as major barriers to seeking MHD and SUD care (Benz et al., 2021; Biancarelli et al., 2019; Cernasev et al., 2021; Lloyd, 2013; Stone, 2015), this study contributes new insights by identifying nuanced, context-specific ways in which stigma and self-protective behaviors shape MHD and SUD care engagement among MMT patients. These findings underscore the need for interventions to address self-stigma surrounding MHD and promote positive coping strategies to prevent substance use as a form of self-medication.

In examining an integrated care model for co-occurring poly-substance use and MHD among MMT patients, this study identified key facilitators, including family members, healthcare staff, and online platforms. Participants highlighted several benefits of integrated care, including improved diagnosis, streamlined referral, and reduced logistic challenges, which are consistent with previous studies (Chuah et al., 2017; Flexhaug, 2012; Glover-Wright et al., 2023; Tran et al., 2022a, b). Across participants’ narratives, family members and healthcare staff emerged as central figures in supporting MHD and SUD care. This aligns with previous studies demonstrating their important roles in first-line diagnosis and linkage to care (Brown et al., 2022; Li et al., 2013; M. X. Nguyen et al., 2019a, b). As in many Asian cultures, Vietnam is a traditionally family-oriented society, where individuals are viewed as embedded members of a larger social system rather than as independent agents (Lin et al., 2011; Thu Trang et al., 2021; N. T. Trang et al., 2025). Family functioning has been associated with better quality of life among MMT patients (Long et al., 2020). However, our study also highlights the need to educate families about MHD and MHD care, as the misconception of MHD being solely linked to substance use could lead to inappropriate or insufficient treatment. Additionally, participants believed that MMT staff were not trained in MHD care, which made them hesitant to share their mental health concerns. Future MHD interventions should prioritize training MMT staff to enhance MHD care. Lastly, in settings where workforce and service capacity for MHD care are limited, the convenience and accessibility of digital platforms become especially valuable. Participants in this study repeatedly highlighted these platforms as a promising opportunity for self-assessment, information-seeking and future MHD intervention. While telehealth shows promise for SUD treatment in low- and middle-income countries (Ojeahere et al., 2022), its use in Vietnam remains limited (Ojeahere et al., 2022; Van Do et al., 2020), underscoring the need for further research on telehealth-based MHD care for MMT patients.

This study is subject to certain limitations. First, the in-depth interviews were exclusively conducted with MMT patients. When this aligns with our primary aim of exploring patient perspectives, future studies should encompass interviews with healthcare providers and caregivers to incorporate other stakeholders’ perspectives on tailoring MHD diagnosis and care. Second, our results were primarily influenced by male voices, indicating a need for future studies to diversify recruitment efforts across genders to achieve a more comprehensive understanding of MHD experiences and identify any distinctive needs and disparities within a broader population.

In summary, significant gaps in MHD care exist among MMT patients in Vietnam, with symptoms often unaddressed until severe, and limited access to MHD specialty treatment. Integrated care models of MHD care in MMT are promising, but future research on feasible models to implement in Vietnam is warranted. Family members, healthcare professionals, and online platforms play significant roles in facilitating MHD continuum of care for MMT patients. Future interventions should focus on education about different types of MHD, dispelling myths surrounding MHD medications, and providing mental health training for MMT staff to improve care quality and reduce stigma.

## Figures and Tables

**Table 1:** Participant characteristics

	*N* = 30	%
**DASS21 depression**		
Severe/moderate	7	23.3
Mild	5	16.7
Normal	18	60.0
**DASS21 anxiety**		
Severe/moderate	16	53.3
Mild	3	10.0
Normal	11	36.7
**DASS 21 stress**		
Severe/moderate	4	13.3
Mild	2	6.7
Normal	24	80.0
**Sex**		
Male	25	83.3
Female	5	16.7
**Age**		
≤ 40 years old	11	53.3
41–49 years old	16	36.7
≥ 50	3	10.0
**Employment**		
Full time	23	77
Part time	4	13
Unemployed	3	10
**Education**		
Less than high school	19	63
Highschool or higher	11	37
**Marital status**		
Married	18	60
Divorced	7	23
Single	5	17
**Clinics**		
Clinic 1	12	40.0
Clinic 2	11	36.7
Clinic 3	7	23.3

**Table 2:** Perceptions on MHD: by society, healthcare staff, and MMT patients

	Society/neighbors	Healthcare staff	MMT patients
**Perspectives on MHD**			
# participants	28	18	18
# quotes	56	18	21
**Stigma**			
# participants	24	1	6
# quotes	42	1	6

*MHD* mental health disorders, *MMT* methadone maintenance treatment

**Table 3:** Experience of MHD, depression, anxiety, and related MHD treatment seeking

	Perceived symptoms	Common perceived symptoms related to MHD	MHD treatment access
Depression	18 participants shared their own depressive symptoms	Alone (“ Mot minh”) Reserved (“khong muon noi chuyen voi ai het”) Disenchanted (“Chan chuong”) Dreary (“Buon chan”) Suicide “ Tu tu” Inexorable “ Khong the vuot qua duoc” Apathetic “ Khong thich gi nua”	3 participants (18%) sought treatment for depression
Anxiety disorders	10 participants shared their own anxiety symptoms	Anxious (“ Lo au”) Immersed (“ Xoay vao day”) Unending (“ Khong dut duoc”) Unfounded (“ Buon lo vo co”) Insomnia (“ Mat ngu”)	No participant sought treatment for anxiety disorders
Stress-related disorders	6 participants highlighted depressive or anxiety symptoms	Persistent insomnia (“Mat ngu trien mien”) Desolate (“suy sup”)	No participant sought for treatment for stress related disorders

*MHD* mental health disorders

## Data Availability

Data is not publicly available due to privacy and confidentiality protections for individuals with substance use disorders.

## References

[R1] Agterberg S, Schubert N, Overington L, & Corace K (2020). Treatment barriers among individuals with co-occurring substance use and mental health problems: Examining gender differences. Journal of Substance Abuse Treatment, 112, 29. 10.1016/j.jsat.2020.01.005 32199543

[R2] Aguirre Velasco A, Cruz ISS, Billings J, Jimenez M, & Rowe S (2020). What are the barriers, facilitators and interventions targeting help-seeking behaviours for common mental health problems in adolescents? A systematic review. BMC Psychiatry. 10.1186/s12888-020-02659-0 PMC7291482 32527236

[R3] Ali MM, Teich JL, & Mutter R (2017). Reasons for not seeking substance use disorder treatment: Variations by health insurance coverage. Journal of Behavioral Health Services & Research. 10.1007/s11414-016-9538-3 27812852

[R4] Benz MB, Cabrera KB, Kline N, Bishop LS, & Palm Reed K (2021). Fear of stigma mediates the relationship between internalized stigma and treatment-seeking among individuals with substance use problems. Substance Use & Misuse. 10.1080/10826084.2021.1899224 33726616

[R5] Biancarelli DL, Biello KB, Childs E, Drainoni M, Salhaney P, Edeza A, Mimiaga MJ, Saitz R, & Bazzi AR (2019). Strategies used by people who inject drugs to avoid stigma in healthcare settings. Drug and Alcohol Dependence. 10.1016/j.drugalcdep.2019.01.037 PMC6521691 30884432

[R6] Braun V, & Clarke V (2022). Thematic analysis: A practical guide. QMiP Bulletin. 10.53841/bpsqmip.2022.1.33.46

[R7] Brown PCM, Dinh TTT, Edsall A, Nguyen TH, Mai PP, Hoffman K, Bart G, Korthuis PT, & Le MG (2022). Familial support in integrated treatment with antiretroviral therapy and medications for opioid use disorder in Vietnam: A qualitative study. Substance Abuse, 43(1), 1004–1010. 35435799 10.1080/08897077.2022.2060435 PMC9678077

[R8] Cacciola JS, Alterman AI, Rutherford MJ, McKay JR, & Mulvaney FD (2001). The relationship of psychiatric comorbidity to treatment outcomes in methadone maintained patients. Drug and Alcohol Dependence, 61(3), 271–280. 11164691 10.1016/s0376-8716(00)00148-4

[R9] California Health Care Foundation. (2021). In their own words: How fragmented care harms people with both mental illness and substance use disorder - California Health Care Foundation. https://www.chcf.org/publication/fragmented-care-harms-people-mental-illness-substance-use-disorder/. Accessed 04/03/2025

[R10] Carpentier PJ, Krabbe PFM, Van Gogh MT, Knapen LJM, Buitelaar JK, & De Jong CAJ (2009). Psychiatric comorbidity reduces quality of life in chronic methadone maintained patients. American Journal on Addictions, 18(6), 470–480. 19874168 10.3109/10550490903205652

[R11] Cernasev A, Hohmeier KC, Frederick K, Jasmin H, & Gatwood J (2021). A systematic literature review of patient perspectives of barriers and facilitators to access, adherence, stigma, and persistence to treatment for substance use disorder. Exploratory Research in Clinical and Social Pharmacy. 10.1016/j.rcsop.2021.100029 PMC9029901 35481114

[R12] Choudhry FR, Mani V, Ming LC, & Khan TM (2016). Beliefs and perception about mental health issues: A meta-synthesis. Neuropsychiatric Disease and Treatment. 10.2147/NDT.S111543 PMC5096745 27826193

[R13] Chuah FLH, Haldane VE, Cervero-Liceras F, Ong SE, Sigfrid LA, Murphy G, Watt N, Balabanova D, Hogarth S, Maimaris W, Otero L, Buse K, McKee M, Piot P, Perel P, & Legido-Quigley H (2017). Interventions and approaches to integrating HIV and mental health services: A systematic review. Health Policy and Planning. 10.1093/heapol/czw169 PMC5886062 29106512

[R14] El Hayek S, Foad W, de Filippis R, Ghosh A, Koukach N, Mahgoub Mohammed Khier A, Pant SB, Padilla V, Ramalho R, Tolba H, & Shalbafan M (2024). Stigma toward substance use disorders: A multinational perspective and call for action. Frontiers in Psychiatry. 10.3389/fpsyt.2024.1295818 PMC10867215 38362033

[R15] Flexhaug M (2012). Integrated models of primary care and mental health & substance use care in the community.

[R16] Giang LM, Trang NT, Diep NB, Thuy DTD, Thuy DT, Hoe HD, Van HTH, Truc TT, Nguyen HH, & Lai NL (2022). An adaptive design to screen, treat, and retain people with opioid use disorders who use methamphetamine in methadone clinics (STAR-OM): Study protocol of a clinical trial. Trials, 23(1), 1–14. 35461300 10.1186/s13063-022-06278-w PMC9034071

[R17] Glover-Wright C, Coupe K, Campbell AC, Keen C, Lawrence P, Kinner SA, & Young JT (2023). Health outcomes and service use patterns associated with co-located outpatient mental health care and alcohol and other drug specialist treatment: A systematic review. Drug and Alcohol Review, 42(5), 1195–1219. 37015828 10.1111/dar.13651 PMC10946517

[R18] Gold AK, Stathopoulou G, & Otto MW (2020). Emotion regulation and motives for illicit drug use in opioid-dependent patients. Cognitive Behaviour Therapy. 10.1080/16506073.2019.1579256 PMC6693992 30760111

[R19] Compton WM Iii, Cottler LB, Jacobs JL, Ben-Abdallah A, & Spitznagel EL (2003). The role of psychiatric disorders in predicting drug dependence treatment outcomes. American Journal of Psychiatry, 160(5), 890–895. 12727692 10.1176/appi.ajp.160.5.890

[R20] Iqbal MN, Levin CJ, & Levin FR (2019). Treatment for substance use disorder with co-occurring mental illness. Focus (San Francisco, Calif.). 10.1176/appi.focus.20180042

[R21] Javed A, Lee C, Zakaria H, Buenaventura RD, Cetkovich-Bakmas M, Duailibi K, Ng B, Ramy H, Saha G, Arifeen S, Elorza PM, Ratnasingham P, & Azeem MW (2021). Reducing the stigma of mental health disorders with a focus on low- and middle-income countries. Asian Journal of Psychiatry. 10.1016/j.ajp.2021.102601 33611083

[R22] Kuek JHL, Raeburn T, & Wand T (2023). Asian perspectives on personal recovery in mental health: A scoping review. Journal of Mental Health. 10.1080/09638237.2020.1818709 32915681

[R23] Lai HMX, Cleary M, Sitharthan T, & Hunt GE (2015). Prevalence of comorbid substance use, anxiety and mood disorders in epidemiological surveys, 1990–2014: A systematic review and meta-analysis. Drug and Alcohol Dependence. 10.1016/j.drugalcdep.2015.05.031 26072219

[R24] Li L, Lin C, Feng N, Nguyen DB, Cao W, Le AT, & Nguyen AT (2020). Stigma related to HIV and drug use: Layers, types, and relations to mental health. AIDS and Behavior. 10.1007/s10461-020-02794-5 PMC7374055 31970581

[R25] Li L, Tuan NA, Liang L-J, Lin C, Farmer SC, & Flore M (2013). Mental health and family relations among people who inject drugs and their family members in Vietnam. International Journal of Drug Policy, 24(6), 545–549. 23910167 10.1016/j.drugpo.2013.06.007 PMC3872211

[R26] Lin C, Pham H, & Hser YI (2023). Mental health service utilization and disparities in the U.S: Observation of the first year into the COVID pandemic. Community Mental Health Journal. 10.1007/s10597-022-01081-y PMC11329229 36609783

[R27] Lin C, Wu Z, & Detels R (2011). Family support, quality of life and concurrent substance use among methadone maintenance therapy clients in China. Public Health. 10.1016/j.puhe.2011.01.009 PMC3112464 21414646

[R28] Lloyd C (2013). The stigmatization of problem drug users: A narrative literature review. Drugs: Education, Prevention and Policy, 20(2), 85–95.

[R29] Long KQ, Thinh OP, Thao TTK, Van Huy N, Lan VTH, Mai VQ, & Van Minh H (2020). Relationship between family functioning and health-related quality of life among methadone maintenance patients: A Bayesian approach. Quality of Life Research. 10.1007/s11136-020-02598-z 32766941

[R30] Mughal AY, Stockton MA, Bui Q, Go V, Pence BW, Ha TV, & Gaynes BN (2021). Examining common mental health disorders in people living with HIV on methadone maintenance therapy in Hanoi, Vietnam. Harm Reduction Journal, 18(1), 1–9. 33892743 10.1186/s12954-021-00495-3 PMC8063421

[R31] Ngoc LB, Hoang VH, Mulvey K, & Rawson RA (2013). Substance use disorders and HIV in Vietnam since Doi Moi (Renovation): An overview. Journal of Food and Drug Analysis, 21(4), S42–S45. 25278736 10.1016/j.jfda.2013.09.032 PMC4179236

[R32] Nguyen LH, Tran BX, Nguyen HLT, Nguyen CT, Hoang CD, Le HQ, Van Nguyen H, Le HT, Tran TD, & Latkin CA (2017). Psychological distress among methadone maintenance patients in Vietnamese mountainous areas. AIDS and Behavior, 21(11), 3228–3237. 28439756 10.1007/s10461-017-1779-5

[R33] Nguyen MX, Go VF, Bui QX, Gaynes BN, & Pence BW (2019). Perceived need, barriers to and facilitators of mental health care among HIV-infected PWID in Hanoi, Vietnam: A qualitative study. Harm Reduction Journal, 16(1), 1–9. 31878934 10.1186/s12954-019-0349-8 PMC6933924

[R34] Nguyen TT, Luong AN, Nham TTT, Chauvin C, Feelemyer J, Nagot N, Jarlais DD, Le MG, & Jauffret-Roustide M (2019). Struggling to achieve a ‘normal life’: A qualitative study of Vietnamese methadone patients. International Journal of Drug Policy. 10.1016/j.drugpo.2019.03.026 PMC6535358 30978641

[R35] Novak P, Feder KA, Ali MM, & Chen J (2019). Behavioral health treatment utilization among individuals with co-occurring opioid use disorder and mental illness: Evidence from a national survey. Journal of Substance Abuse Treatment, 98, 47. 10.1016/j.jsat.2018.12.006 30665603 PMC6350939

[R36] Ojeahere MI, Kiburi SK, Agbo P, Kumar R, & Jaguga F (2022). Telehealth interventions for substance use disorders in low- and- middle income countries: A scoping review. PLoS Digital Health. 10.1371/journal.pdig.0000125 PMC9931245 36812539

[R37] Oviedo E, Means RF, Lilley-Haughey K, & Hua LL (2023). Integration of substance use disorder treatment into a traditional community mental health treatment system. Psychiatric Services. 10.1176/appi.ps.202100643 36695014

[R38] Richardson A, Richard L, Gunter K, Cunningham R, Hamer H, Lockett H, Wyeth E, Stokes T, Burke M, & Green M (2020). A systematic scoping review of interventions to integrate physical and mental healthcare for people with serious mental illness and substance use disorders. Journal of Psychiatric Research, 128, 52–67. 32521251 10.1016/j.jpsychires.2020.05.021

[R39] Silva TC, & Andersson FB (2021). The “black box” of treatment: Patients’ perspective on what works in opioid maintenance treatment for opioid dependence. Substance Abuse: Treatment, Prevention, and Policy. 10.1186/s13011-021-00378-7 PMC8111936 33971909

[R40] Stone R (2015). Pregnant women and substance use: Fear, stigma, and barriers to care. Health & Justice. 10.1186/s40352-015-0015-5

[R41] Strain EC (2002). Assessment and treatment of comorbid psychiatric disorders in opioid-dependent patients. The Clinical Journal of Pain, 18(4), S14–S27. 12479251 10.1097/00002508-200207001-00003

[R42] Thu Trang N, Jauffret-Roustide M, Minh Giang L, & Visier L (2021). How to be self-reliant in a stigmatising context? Challenges facing people who inject drugs in Vietnam. International Journal of Drug Policy, 87, Article 102913. 10.1016/j.drugpo.2020.102913 PMC8118722 32855011

[R43] Tran HV, Filipowicz TR, Landrum KR, Nong HTT, Tran TTT, Pence BW, Go VF, Le GM, Nguyen MX, Verhey R, Chibanda D, Ho HT, & Gaynes BN (2022). Stigma experienced by people living with HIV who are on methadone maintenance treatment and have symptoms of common mental disorders in Hanoi, Vietnam: A qualitative study. AIDS Research and Therapy. 10.1186/s12981-022-00491-y PMC9753276 36517849

[R44] Tran HV, Nong HTT, Tran TTT, Filipowicz TR, Landrum KR, Pence BW, Le GM, Nguyen MX, Chibanda D, & Verhey R (2022). Adaptation of a problem-solving program (friendship bench) to treat common mental disorders among people living with HIV and AIDS and on methadone maintenance treatment in Vietnam: Formative study. JMIR Formative Research, 6(7), e37211. 35802402 10.2196/37211 PMC9308082

[R45] Tran TD, Tran T, & Fisher J (2012). Validation of three psychometric instruments for screening for perinatal common mental disorders in men in the north of Vietnam. Journal of Affective Disorders, 136(1–2), 104–109. 21907417 10.1016/j.jad.2011.08.012

[R46] Trang K, Ly AT, Lam LX, Brown CA, To MQ, Sullivan PS, Worthman CM, Giang LM, & Jovanovic T (2021). Mental health in HIV prevention and care: A qualitative study of challenges and facilitators to integration in Vietnam. Social Science & Medicine. 10.1016/j.socscimed.2021.113978 PMC8684791 34000583

[R47] Trang NT, Hoe HD, Anh NH, Thuy DTT, & Bart G (2025). When she’s there, I no longer worry about her being arrested”—Family perspectives on compulsory drug rehabilitation in Vietnam and implications for community-based services. International Journal of Drug Policy, 135, 104681. 39719810 10.1016/j.drugpo.2024.104681 PMC11748025

[R48] Valentijn PP, Schepman SM, Opheij W, & Bruijnzeels MA (2013). Understanding integrated care: A comprehensive conceptual framework based on the integrative functions of primary care. International Journal of Integrated Care, 13(JANUARY-MARCH 2). 10.5334/ijic.886 PMC3653278 23687482

[R49] Van Do V, Spears CA, Van Minh H, Huang J, Redmon PB, Long NX, & Eriksen MP (2020). Perceptions about mindfulness and text messaging for smoking cessation in Vietnam: Results from a qualitative study. JMIR Mhealth and Uhealth, 8(6), e17337. 32442140 10.2196/17337 PMC7381024

[R50] Vuong DA, Van Ginneken E, Morris J, Ha ST, & Busse R (2011). Mental health in Vietnam: Burden of disease and availability of services. Asian Journal of Psychiatry, 4(1), 65–70. 23050918 10.1016/j.ajp.2011.01.005

[R51] WHO. (2015). Global health workforce, finances remain low for mental health. https://www.who.int/news/item/14-07-2015-global-health-workforce-finances-remain-low-for-mental-health. Accessed 04/03/2025

[R52] Zhu Y, Mooney LJ, Yoo C, Evans EA, Kelleghan A, Saxon AJ, Curtis ME, & Hser YI (2021). Psychiatric comorbidity and treatment outcomes in patients with opioid use disorder: Results from a multisite trial of buprenorphine-naloxone and methadone. Drug and Alcohol Dependence. 10.1016/j.drugalcdep.2021.108996 PMC8674982 34555691

